# Transcriptional Analyses of Acute Exposure to Methylmercury on Erythrocytes of Loggerhead Sea Turtle

**DOI:** 10.3390/toxics9040070

**Published:** 2021-03-29

**Authors:** Javier Hernández-Fernández, Andrés Pinzón-Velasco, Ellie Anne López, Pilar Rodríguez-Becerra, Leonardo Mariño-Ramírez

**Affiliations:** 1 Department of Natural and Environmental Science, Marine Biology Program, Faculty of Science and Engineering, Genetics, Molecular Biology and Bioinformatic Research Group–GENBIMOL, Jorge Tadeo Lozano University, Cra. 4 No 22-61, Bogotá 110311, Colombia; mariad.rodriguezb@utadeo.edu.co; 2 Faculty of Sciences, Department of Biology, Pontificia Universidad Javeriana, Calle 45, Cra. 7, Bogotá 110231, Colombia; 3 Bioinformática y Biología de Sistemas, Universidad Nacional de Colombia, Calle 45, Cra. 30, Bogotá 111321, Colombia; ampinzonv@unal.edu.co; 4 IDEASA Research Group-Environment and Sustainability, Institute of Environmental Studies and Services, Sergio Arboleda University, Bogotá 111711, Colombia; ellie.lopez@usa.edu.co; 5 NCBI, NLM, NIH Computational Biology Branch, Building 38A, Room 6S614M 8600 Rockville Pike, MSC 6075, Bethesda, MD 20894-6075, USA; marino@ncbi.nlm.nih.gov

**Keywords:** *Caretta caretta*, RNA-seq, differential expression, methylmercury, transcriptomics

## Abstract

To understand changes in enzyme activity and gene expression as biomarkers of exposure to methylmercury, we exposed loggerhead turtle erythrocytes (RBCs) to concentrations of 0, 1, and 5 mg L^−1^ of MeHg and de novo transcriptome were assembled using RNA-seq. The analysis of differentially expressed genes (DEGs) indicated that 79 unique genes were dysregulated (39 upregulated and 44 downregulated genes). The results showed that MeHg altered gene expression patterns as a response to the cellular stress produced, reflected in cell cycle regulation, lysosomal activity, autophagy, calcium regulation, mitochondrial regulation, apoptosis, and regulation of transcription and translation. The analysis of DEGs showed a low response of the antioxidant machinery to MeHg, evidenced by the fact that genes of early response to oxidative stress were not dysregulated. The RBCs maintained a constitutive expression of proteins that represented a good part of the defense against reactive oxygen species (ROS) induced by MeHg.

## 1. Introduction

Mercury (Hg) represents one of the most serious environmental threats to wildlife well-being [1,2]. It is among the most toxic and persistent pollutants released into marine ecosystems [3]. Furthermore, it represents a greater problem in aquatic systems, where inorganic Hg is transformed into organic Hg (methylmercury, MeHg), which is biomagnified in the trophic chain [1,2,4].

In the sea, MeHg is a molecule of great concern to marine populations as it bioaccumulates and biomagnifies in tissues, causing health damage [5]. MeHg makes up 75–99% of the total mercury (Hg-T) detected in living organisms [6], and is much more toxic than inorganic mercury [7].

MeHg is a weak electrophile with a strong affinity for sulfhydryl groups (-SH) and selenoles (-SeH), which are functional in proteins containing cysteines and selenocysteines respectively, with consequences such as (i) inactivation of molecules related to electrophilic forms, (ii) decrease in Se availability and, therefore, selenoprotein synthesis, and (iii) increase in intracellular MeHg concentration [8,9].

Exposure to MeHg has been extensively studied and is well documented in mammals, birds, and fish and includes neurotoxicity, impaired growth and development, reduced reproductive success, liver and kidney damage, and immunomodulation [2,10,11,12,13,14]. Among other changes associated with exposure to MeHg are gene expression levels linked to oxidative stress, microtubule disruption, mitochondrial dysfunction, endoplasmic reticulum stress, disruption of intracellular calcium levels, programmed cell death, dysregulation of transcription and translation, expression of heat shock proteins, and production of reactive oxygen species (ROS) [2,14,15,16,17]. However, the effects on reptiles remain poorly understood [7,18,19,20,21]. Studying these early stages of mercury damage is the basis for identifying metabolic pathways that are activated in response to mercury contamination.

Advances in next-generation sequencing technologies, such as massive RNA sequencing (RNA-seq), allow for the characterization of molecular responses to stimuli with impressive sensitivity for non-model organisms, such as the loggerhead turtle [22,23,24]. The comparison of genes that are differentially expressed in erythrocytes (RBCs) exposed to a xenobiotic, such as methylmercury, allows for the identification of biomarkers for stress and adaptive response [25]. It is very important to perform differential expression analysis at very low levels of MeHg to identify possible “expression signatures”. These expression signatures could be used as early markers of exposure to MeHg, before detecting physiological or biochemical changes (Oliveira et al., 2020). Exposure of RBCs to low doses of MeHg (1–5 ppm) for a short time (a few hours) provides the opportunity to detect a possible compensatory hormonal mechanism, which can be interpreted as molecular responses that result in cellular protection [26].

Studies carried out on the toxicity of MeHg in wild animals and humans using next-generation sequencing (RNA-seq) have generated knowledge about the molecular mechanisms of this compound produced in different tissues [26,27,28,29,30,31]. The cellular and molecular mechanisms that produce MeHg-mediated toxicity are not yet fully understood, and there are species for which very little is known (i.e., sea turtles) [4]. However, these types of studies have not been performed in loggerhead turtles. These reptiles are long-lived and carry out extensive migrations, characteristics that allow them to accumulate Hg for a long time. For these reasons, they are sentinel species: bioindicators of pollution in the marine environment [32]. These marine chelonians are in population decline due to anthropic and environmental influences, appearing on the red list of threatened species of the International Union for Conservation of Nature (IUCN, 2019-2) as critically endangered (www.iucnredlist.org, accessed on 13 January 2021). For this reason, it is important to propose alternative study methodologies that do not compromise the life of these animals that are instrumental for the health of marine ecosystems [32]. Recently, cell cultures of different tissues of sea turtles have been established to allow research in toxicology [33]. These cells are being used in in vitro tests, producing a frame of reference on the initial response, with the effects of different xenobiotics evaluated [33,34,35,36,37]. The nucleated RBCs of sea turtles represent a model for in vivo ecotoxicology research to reveal the effects of Hg and other heavy metals and environmental pollutants [32].

In previous studies, we demonstrated that an increase in the enzymatic activity of Glutathione S transferase and superoxide dismutase, as well as the production of malonaldehyde, was revealed when loggerhead turtle RBCs were exposed to 0, 1, and 5 mg L^−1^ of MeHg for 12 h. The observed response shows the increase in reactive oxygen species in RBCs [32]. In this study, we set out to perform an analysis of the global transcriptional profiles in RBCs of the loggerhead turtle, *Caretta caretta*, to identify cellular pathways or biological processes affected by MeHg while preliminarily elucidating the molecular mechanisms of toxicity used by this xenobiotic against this marine turtle.

## 2. Materials and Methods

### 2.1. Permits to Collect Samples

The samples and methods used in this study followed the ethical standards of Colombian legislation and those of the ethics committee of the Jorge Tadeo Lozano University (UJTL) (Project 340-07-10). The project had a permit to collect samples of Colombian biodiversity, granted to the UJTL by the Ministry of the Environment (Resolution 1271 of 23 October 2014, IDB040I).

### 2.2. Study Area and Sample Collection

Peripheral blood was collected from five captive, pre-juvenile loggerhead turtles at the El Rodadero Aquarium in Santa Marta (11°1301.09″ N, 74°14013.75″ W). The details of blood sample collection and RBC isolation have been described previously [32]. In brief, blood samples were extracted from the dorsal region of the cervical sinus. For the collection of peripheral blood, we used 4 mL sterile syringes and 2 mL tubes with RNAlater (Ambion Inc., Austin, TX, USA). The samples were transported on dry ice to the Molecular Biology laboratory at the Jorge Tadeo Lozano University, Bogotá.

### 2.3. Experimental Design

**Figure 1:**
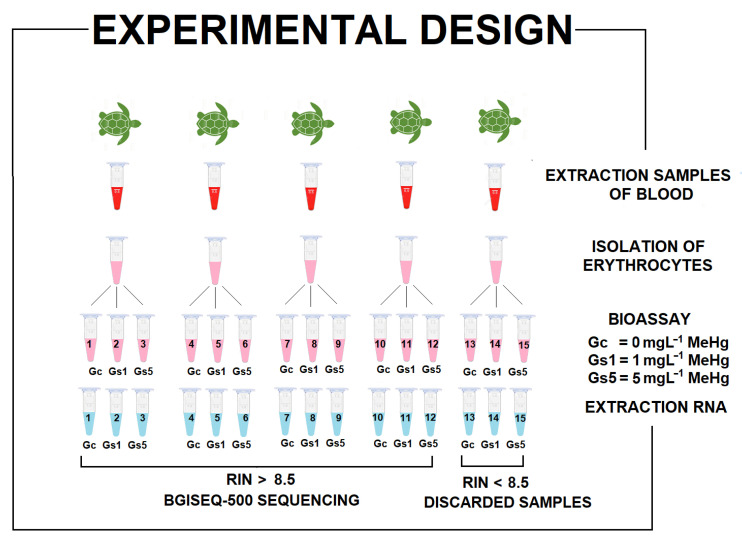
Experimental design. Five steps were carried out prior to sequencing by RNA-seq: (1) samples were obtained from five *Caretta caretta* turtles; (2) erythrocytes were isolated; (3) the bioassay was carried out; (4) RNA was extracted; and (5) samples were selected for sequencing according to the RNA integrity number (RIN).

Aliquots of 2 × 10^6^ RBCs/ml from five peripheral blood samples were isolated by centrifugation and then resuspended in 1.5 mL Eppendorf tubes in MEM-S medium [32]. We exposed the RBCs to doses of 0, 1, and 5 mg L^−1^ of methylmercury II chloride (CH_3_HgCl) (Merck KGaA, Darmstadt, Germany) for 12 h at 30 °C. Each of the five samples was exposed to the three concentrations of CH_3_HgCl (MeHg), thus producing 15 samples altogether. As a control, we used RBCs in a culture medium with sterile saline solution (used to dilute the MeHgCl) (Figure 1).

### 2.4. RNA Extraction, Library Construction and Sequencing

At 12 h of incubation, the RBCs exposed to MeHg were washed twice with 0.9% sterile saline solution (Baxter Healthcare Corporation, Deerfield, IL, USA), then centrifuged at 2500 rpm at 22 °C, resuspended in 15 volumes of RNAlater^®^ (Ambion, Inc., Austin, TX, USA), and stored at 4 °C for one week.

Before extraction, the RNAlater was removed and the RBCs were washed with 1 volume of 0.9% sterile saline solution (4 °C), then centrifuged at 5000× *g* for 10 min at 22 °C. We extracted total RNA from each of the samples (~25 µL RBCs/mL) using RNeasy^®^ Mini Extraction Kit, following the instructions of the manufacturer (Quiagen, Hilden, Germany). The extracted RNAs were treated with DNase according to the instructions of the manufacturer (RNase-Free DNase Set, Cat No / ID: 79254) (Quiagen, Hiden, Germany) and diluted in RNAsa-free MilliQ water. RNA was stored at −80 °C for subsequent evaluations.

The RNA was quantified and its quality analyzed by spectrophotometry, using a Nanodrop-1000 (Thermo Scientific, Wilmington, DE, USA). The RNA was evaluated by electrophoresis in 1% agarose gels (100 V, 30 min). Each of the total RNA preparations was individually assessed for RNA quality based on the 28S/18S ratio and the RNA integrity number (RIN) was measured by 4200 TapeStation capillary electrophoreses (Agilent Technologies, Waldbronn, Germany) at the Research Center of the Colombian Agricultural Research Corporation in Tibaitatá, Cundinamarca (Agrosavia, Bogotá, Colombia). Samples with RIN > 8.5 were selected for further analysis (Figure S1). The Beijing Genomics Institute (BGI, Shenzhen, China) carried out the fragmentation of mRNA, cDNA libraries construction, and sequencing using the BGISEQ-500 platform, following the previously published methodology [38].

### 2.5. Assembly of the Transcriptome

We evaluated the raw sequencing data using the FastQC tool [39]. Then, the adapters and the low-quality reads were filtered with Trimomatic [40]. We used the Trinity pipeline v2.0.6 to assemble each of the individual samples, the three experimental groups (0 = control; 1 mg L^−1^ = Gs1 and 5 mg L^−1^ = Gs5), and the union of all samples, using default parameters [41]. We evaluated the assembled transcripts for their integrity and quality with the Dogma v-3.4 program (Domain-Based General Measure for Transcriptome and Proteome Quality Assessment) [42], comparing them to the conserved protein domains and domain arrangements of eukaryotes and vertebrates.

### 2.6. Unigenes Prediction and Functional Annotation

We grouped the obtained transcripts in pairs according to the similarity of the sequences to produce longer and more complete consensus sequences using the TGICL v2.0.6 program (a software system for fast clustering of large EST datasets) [43]. The resulting sequences (unigenes) were classified into two groups: clusters (CL, transcript groups with a similarity higher than 70%), and singletons. Clusters and singletons were annotated with BlastN and BlastX v2.2.23 against the Nt, Nr, KOG, and KEGG databases with default parameters, except for the E-value (modified to 1e^−5^) [44]. Later, the unigenes were annotated to the GO database using SwissProt and the Blast2GO v2.5.0 program with default parameters [45]. We used WEGO software v2.0 [46] to determine the GO functional classifications and evaluate the distribution of functions of unigenes. Lastly, we used InterProScan5 v5.11-51.0 with default parameters to predict protein families, domains, and functional sites [47]. Unigenes were also used to predict the coding regions (CDS) and open reading frames (ORFs) using the Transdecoder v3.0.1 program with default parameters [48].

### 2.7. Identification and Annotation of Differentially Expressed Genes

We mapped the clean reads to the unigenes obtained previously using Bowtie2 v2.2.5 [49] and calculated the expression level of each of the genes using RSEM v1.2.12 [50]. The expression levels of the unigenes were measured in fragments per kilobase of exon per million reads mapped (FPKM), which normalizes the paired transcript and reads counts both for their length and for the total number of reads mapped in the sample [51]. We identified differentially expressed genes using the DEseq2 program [52] (significant DEGs: log2 fold change ≥ 1 or log2 fold change ≤ −1, and Adjusted *p* value ≤ 0.05), which normalizes the data taking into account the differences in the total number of reads per library. Further, we used PossionDis (significant DEGs: fold change ≥ 1 or fold change ≤ −1, a FDR ≤ 0.001). 

The DEGs were identified by comparing the control group (Gc) with each of the groups exposed to MeHg (Gs1 and Gs5), and comparing the two exposed groups with each other (Gs1 versus Gs5). Subsequently, the distribution of the expressed genes was graphed using heat maps and MA plots with the heatmap and ggmaplot (DEseq2) functions, respectively. In the heat maps, we divided the expression levels of the DEGs into orange and blue regions that indicated high and low levels of relative expression, respectively. We compared the set of differentially expressed genes shared between the different treatments with Venn diagrams using the Venny 2.0 program [53].

Differentially expressed genes were mapped to GO terms and the KEGG database to perform functional enrichment and metabolic pathway analyses, respectively. We then calculated the false discovery rate for each p-value. Terms whose FDR was less than 0.01 were defined as having significant enrichment.

### 2.8. Statistical Analysis of the Correlation between the Relative Expression (FPKM) of the Genes GST, Cu/Zinc-SOD, Mn-SOD, and Tbxas1 with the Enzymatic Activity of GST and SOD, and the Amount of MDA (µM) Produced by Lipid Peroxidation

In a previous experiment, we used the same RBC samples [32] as in this study to determine the activities of the Cu/Zinc-SOD, Mn-SOD, GST enzymes, and the amount of MDA produced. To verify the correlation between the relative expression (FPKM) of the GST, Cu/Zinc-SOD, Mn-SOD, and Tbxas1 genes and the enzymatic activities [32], we used Pearson’s multiple correlation analysis using the StatR v.1.8 statistical packages designed for Rwizard v.4.3 [54] The mapped read count measures the relative expression level of the genes and statistical methods are then applied to test the significance of the differences between groups [55]. This approach of using FPKM as a measure of relative expression has been used previously [29,56,57,58].

As dependent variables in each of the analyses, the following were selected: GST activity, Cu/Zinc-SOD activity, Mn-SOD, and MDA concentration (µM), respectively. As independent variables, the following were selected: relative expression (GST, SOD/Cu/Zinc, Mn-SOD, and Tbxas1 (FPKM)), the initial total concentration of MeHg, and concentration of MeHg added to each individual. The Tbxas1 gene was correlated with the concentration of MDA because this gene participates in the isomerization of the PGH2 prostaglandin, transforming it into 12-hydroxy-5, 8, 10-heptatrienic acid (12-HHT), and malondialdehyde (MDA).

### 2.9. Data Availability

All data are available at NCBI under project accession number PRJNA575050. The raw sequences data of RNA-seq in FASTQ format were deposited at the NCBI in the Sequence Read Archive Database (SRA) under the accession numbers: SRR10412101, SRR10412100, SRR10412099, SRR10412098, SRR10412097, SRR10412096, SRR10412095, SRR10412094, SRR10412093, SRR10412092, SRR1041209, and SRR10412090.

## 3. Results

### 3.1. Sequencing, Filtering of Readings, and Assembly

The sequences of the 12 libraries presented high quality according to the FASTQC analysis (three samples, T13, T14, and T15 were removed as they presented a RIN less than 7,5). However, sequencing reads containing adapter contamination, and unknown bases (N), were removed (they represented 1.8% of the total readings) (Table S1). We assembled all individual transcripts (T1 to T12). Then, we assembled the transcriptomes of Gc (T1, T4, T7, and T10), Gs1 (T2, T5, T8, and T11), and Gs5 (T3, T6, T9, and T12). Lastly, we assembled the composite transcriptome, which included the readings of all 12 individuals. The metrics for each of the assemblies are presented in Table 1.

The de novo assembly of the twelve individuals produced a composite transcriptome with 121,933 unigenes, with an average length of 1444 bp, N50 of 3520, and a GC percentage of 46.98% (Table 2). The size of the unigenes ranged between 300 and 3000 bp (Figure S2). The 12 samples had on average 72.2% of their transcripts matching with the composite transcriptome, while the partial transcriptomes of Gc, Gs1, and Gs5 showed 99.8% of transcripts matching with the composite transcriptome (Table 1). We used the DOGMA web server to evaluate the level of integrity of the transcriptome. We identified 76.6% and 96.8% of the central assemblages of vertebrates and eukaryotes, respectively. These results support the high quality and integrity of our composite loggerhead transcriptome. Furthermore, these results are in agreement with those published in previous studies [59,60,61].

### 3.2. Functional Annotation

**Figure 2:**
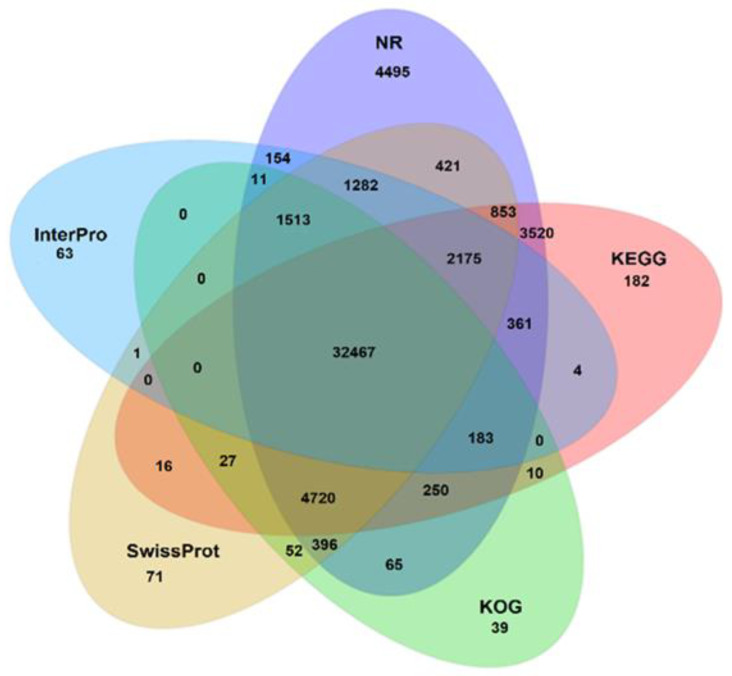
Venn diagram showing the genes of the loggerhead turtle, *Caretta caretta*, annotated to the databases Nr, KOG, KEGG, Swissprot, and Interpro.

We generated unigenes for each of the assembled transcriptomes. The composite transcriptome annotation produced 72,700 (59.62%) unigenes with significant agreement against at least one of the analyzed databases (Table 2). A total of 11,693 (9.6%) and 32,467 (44.8%) unigenes were annotated to 7 and 5 databases respectively (Table 2, Figure 2). Our composite transcriptome was also scored against Testudines proteomes (*Chelonia mydas*, *Pelodiscus sinensis*, and *Chrysemys picta belli*), and against a previously published *Caretta caretta* transcriptome (Genbank PRJNA560561), obtaining significant agreement against 97,546 proteins (80.5%) and 110,846 unigenes (90.9%), respectively.

### 3.3. Differential Gene Expression

**Figure 3:**
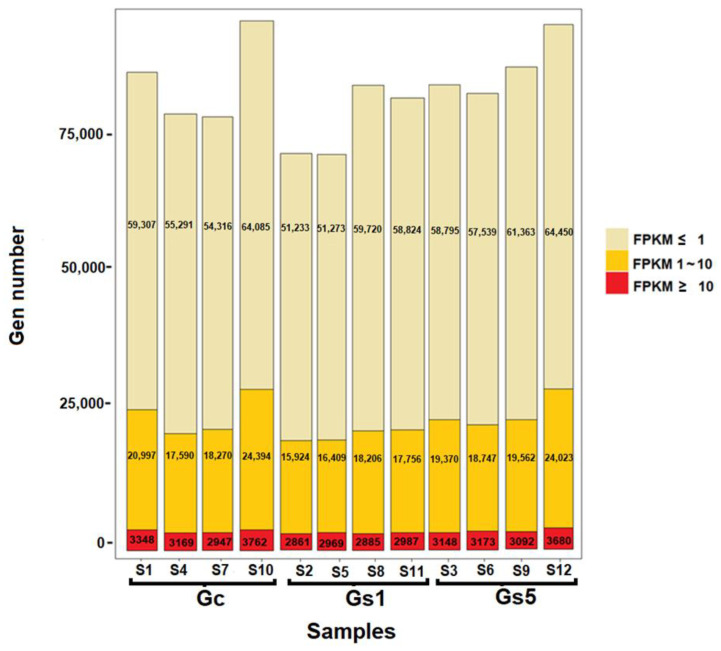
Quantification of transcripts of the loggerhead turtle, *Caretta caretta*, for each of the samples evaluated according to their high (FPKM > 10), medium (FPKM between 1–10), and low (FPKM < 1) abundance.

To evaluate the expression characteristics of the mRNA, we estimated the relative expression levels (FPKM) of each of the samples for each transcript (Figure 3). We classified the expression of genes into three categories: (i) genes that presented low relative expression levels (FPKM < 1), which represented the majority of genes (72%); (ii) genes that presented medium relative expression levels (FPKM between 1–10), which represented a quarter of the genes (24%); and (iii) genes that presented high levels of relative expression (FPKM > 10), which represented a minority of the genes (4%).

On average, each of the 12 samples expressed 80,453 genes. No significant differences were found between the number of genes expressed between Gc, Gs1, and Gs5 (Kruskal–Wallis, *p* > 0.05). It is important to note that when Gs1 was compared to Gs5, it showed a marked reduction in gene expression (5274, 3352, and 348 fewer genes respectively expressed in categories i, ii, and iii described above) in all three categories (Figure 3). On the contrary, when Gc was compared with Gs5, it presented smaller differences in the expression of genes in the first two categories (2287 and 118 fewer genes than Gs5, respectively), and in the third category, Gc expressed more genes than Gs5 (33 genes) (Figure 3). The results appear to show that low concentrations of MeHg (1 mg L^−1^) generally decrease gene expression while higher concentrations increase it (5 mg L^−1^).

**Figure 4:**
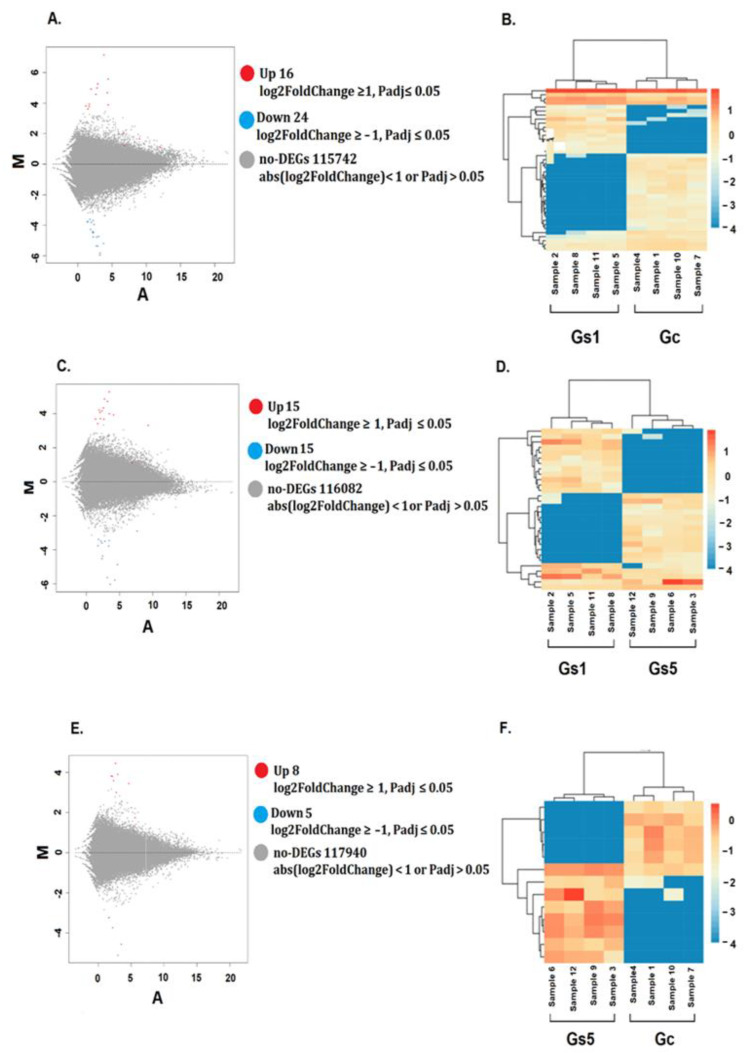
MA plots and heat maps representing the differentially expressed genes in the transcriptome of the loggerhead turtle, *Caretta caretta*. (**A**,**B**) Gc vs. Gs1, (**C**,**D**) Gs1 vs. Gs5, (**E**,**F**) Gc vs. Gs5.

**Figure 5:**
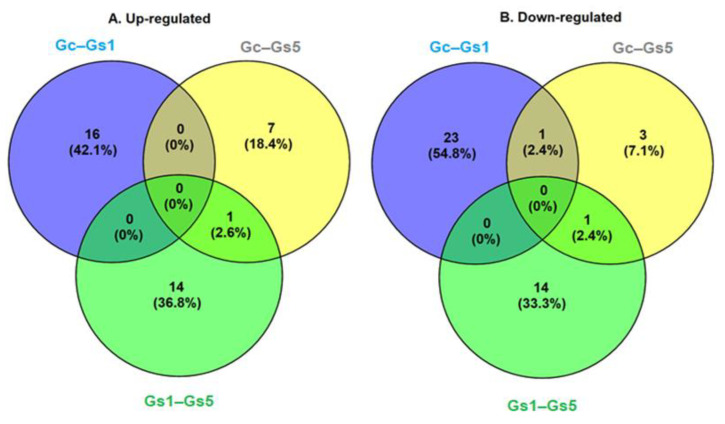
Venn diagrams indicating the number of genes differentially expressed in the transcriptome of the loggerhead turtle, *Caretta caretta*. (**A**) Upregulated genes and (**B**) downregulated genes in erythrocytes exposed in vitro to doses of 0, (Gc), 1 (Gs1), and 5 (Gs5) mg L^−1^ of MeHg for 12 h.

The evaluation of dysregulation caused by MeHg to the RBCs showed that 83 genes produced significant differential gene expression (log2 fold change ≥ 1, or log2 fold chance ≤ −1, Padj ≤ 0.05 and FDR ≤ 0.001) between the Gc–Gs1, Gc–Gs5, and Gs1–Gs5 treatments. The group of upregulated genes under MeHg stress produced 39 matches and the downregulated genes produced 44 matches. These differentially expressed genes were annotated to the databases as follows: Nt (79 genes, 95.2%), Nr (80 genes, 96.3%), SwissProt (78 genes, 94%), KEGG (74 genes, 89.1%), KOG (69 genes, 83.1%), InterPro (73 genes, 87.9%) and GO (27 genes, 32.5%). In summary, only 80 genes produced significant matches and were annotated to at least one database. The other three genes had no matches and could not be annotated (NC). Of the 83 dysregulated genes, four genes were in more than one comparison. This means we identified a total of 79 unique DEGs (Figure 4 and Figure 5).

When the gene expression of the Gc and Gs1 treatments were compared, we found 16 upregulated genes (between 1 and 7 fold) and 24 downregulated genes (between −1.7 and −5 fold) (Figure 4A,B) (Table S2). The comparison between the Gs1 and Gs5 treatments showed 15 upregulated genes (a fold change between one and more than five) and 15 downregulated genes (a fold change between −1.7 and more than −6) (Figure 4C,D) (Table S3). Lastly, between Gc and Gs5 we found eight upregulated (between 1 and 5 fold) and five downregulated (between −3 and −5 fold) genes (Figure 4E,F; Figure S3A–C, Table S4).

We found that none of the upregulated and downregulated genes were common to all three established relationships (Figure 5). Of the 39 upregulated genes, 1 gene was common between the Gc–Gs5 and Gs1–Gs5 comparisons. A total of 16, 14, and 7 genes were unique in the Gc–Gs1, Gs1–Gs5, and Gc–Gs5 comparisons, respectively (Figure 5). Of the 44 downregulated genes, two genes were common: one between the Gc–Gs1 and Gc–Gs5 comparisons, and one between the Gs1–Gs5 and Gc–Gs5 comparisons. A total of 23, 14, and 3 unigenes were unique among the comparisons Gc–Gs1, Gs1–Gs5 and Gc–Gs5, respectively (Figure 5).

### 3.4. Functional Enrichment Analysis with GO Terms of Differentially Expressed Genes

**Figure 6:**
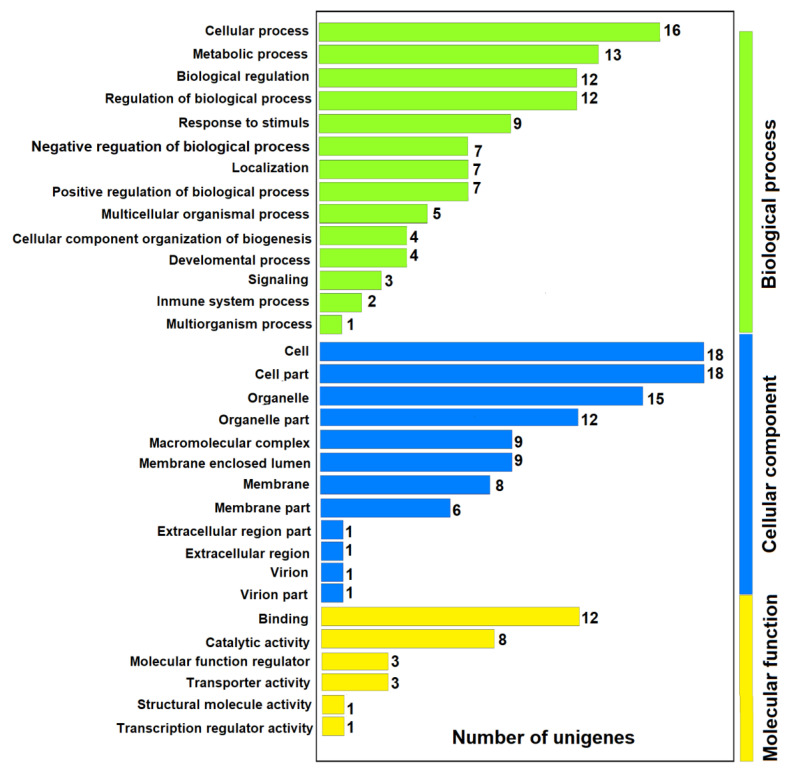
Distribution by categories of the GO classification of the 83 differentially expressed genes (DEGs). The number of unigenes in each category is shown.

The enrichment of GO terms of DEGs was carried out to identify the related biological processes and the functions affected by MeHg in the RBCs. The GOs were assigned to the three gene ontologies: biological processes, cellular components, and molecular function. Of the 79 DEGs, only 27 were annotated against the GO database (11 upregulated genes and 16 downregulated genes). When Gc was compared with Gs1, 113 unigenes were classified into 28 GO functional group categories (14 in biological processes, 10 in cellular components, and 4 in molecular function). When comparing Gs1 with Gs5, 103 unigenes were classified into 27 GO functional group categories (13 in biological processes, 10 in cellular components and 4 in molecular function), and finally, when comparing Gc with Gs5, 17 unigenes were classified into 17 GO functional group categories (eight in biological processes, six in cellular components and three in molecular function) (Figure 6).

To acquire an understanding of what may be happening at the cellular level between Gc, Gs1, and Gs5, the annotation was completed manually using the Uniprot and Gene Ontology databases. The differentially expressed unigenes in the three comparisons were classified according to molecular function in the following categories: oxidative stress, regulation of the cell cycle, signaling, regulation of transcription and translation, binding to metal ions, autophagy, apoptotic processes, metabolic processes, membrane transport, DNA repair, mitochondria, and miscellaneous. The results of the top 25 annotations are presented in Table 3 (all annotation results are presented in Table S5).

The DEGs between Gc and Gs1 presented 40 dysregulated unigenes which were grouped into 11 GO categories according to their function. The most representative categories were: regulation of transcription (two upregulated and ten downregulated genes), signaling (one upregulated and seven downregulated genes), oxidative stress (three upregulated and three downregulated genes), metabolic processes (three upregulated and two downregulated genes), and binding to metal ions (four upregulated genes) (Table S2). The DEGs between Gs1 and Gs5 presented 30 dysregulated unigenes which were grouped into 11 GO categories according to their function. The most representative categories were: regulation of transcription (three upregulated and four downregulated genes), signaling (three upregulated and three downregulated genes), oxidative stress (three upregulated and one downregulated gene(s)), transmembrane transport activity (two upregulated and two downregulated genes), and binding to metal ions (three upregulated genes) (Table S3). The DEGs between Gc and Gs5 presented 13 dysregulated unigenes, which were grouped into six GO categories according to their function. The two most representative categories were: regulation of transcription (three upregulated genes) and binding to metal ions (three downregulated genes) (Table S4).

### 3.5. Analysis of Functional Enrichment of KEGG Pathways of Differentially Expressed Genes

**Figure 7:**
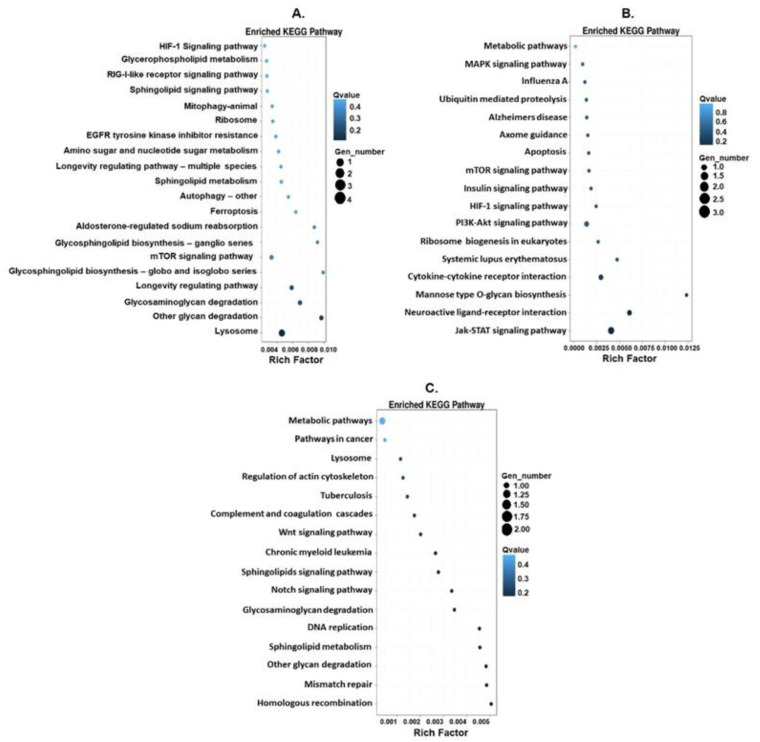
KEGG pathway functional enrichment of DEGs. The x-axis shows the enrichment factor. The y-axis shows pathway name. Point size indicates DEG number (the bigger dots refer to larger amounts); the larger the value, the more significant the enrichment. The color represents the q-value (high: white, low: blue), a lower q-value indicates a more significant enrichment. (**A**) Comparison between Gc and Gs1, (**B**) comparison between Gs1 and Gs5, (**C**) comparison between Gc and Gs5.

The enrichment analysis of KEGG pathways carried out on differentially expressed genes showed that, when comparing Gc to Gs1 (Figure 7A), Gs1 to Gs5 (Figure 7B), and Gc to Gs5 (Figure 7C), the genes were associated with 20, 17, and 16 pathways, respectively. In the comparison between Gc–Gs1 and Gs1–Gs5, we found that the pathways with the highest enrichment were the lysosome (four genes) and the Jak-STAT (three genes) pathways, pathways that participate in the regulation of the response to oxidative stress mediated by MeHg [62,63]. In the comparison between Gc–Gs1, Gs1–Gs5, and Gc–Gs5, genes that participate in sphingolipid metabolism, autophagy, apoptosis, the HIF signaling pathway, mTOR, and PI3K-Akt were also enriched, which have been reported to be upregulated or downregulated by MeHg [64,65,66,67] (Figure 7).

### 3.6. Correlation between the Relative Expression (FPKM) of the GST, SOD, and Tbxas1 Genes (RNA-seq Data) with the Enzymatic Activity of GST, SOD, and the Amount of MDA (μM) Produced

**Figure 8:**
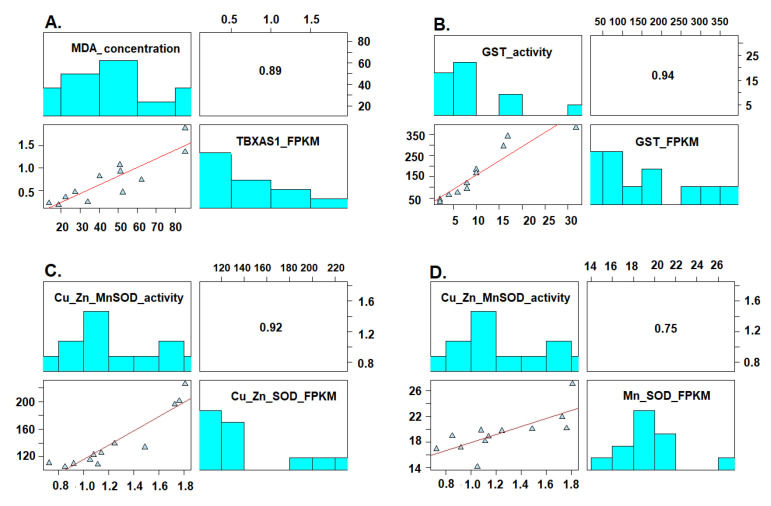
Correlation between the enzymatic activity of GST, SOD, and the concentration of MDA (μM) produced by lipid peroxidation with the relative expression (FPKM) of the genes in the loggerhead turtle, exposed to MeHg. (**A**) TBXAS1 with MDA (µM), (**B**) GST with GST activity, (**C**) Cu/Zn-SOD with Cu/Zn-SOD activity, (**D**) Mn-SOD with Mn-SOD activity.

We found, based on the multiple correlation analyses carried out on the Cu/Zinc-SOD, Mn-SOD, and GST activities and the concentration of MDA, that in the three cases the activity of these enzymes (SOD and GST) and the concentration of MDA were directly correlated with the relative expression of the Cu/Zinc-SOD, Mn-SOD, GST, and Tbxas1 genes (*R* = 0.92, 0.75, 0.94 and 0.89, respectively) (*p* < 0.001) (Figure 8A–D). Additionally, we observed that the activity of the Mn-SOD enzyme and its relative expression were correlated with the concentration of added MeHg, *R* = 0.75 (*p* = 0.005) and *R* = 0.67 (*p* = 0.016), respectively (Figure S4A–D).

## 4. Discussion

The results of our study provide information on the differential expression of genes in loggerhead sea turtle RBCs exposed to MeHg. It should be noted that the information available on non-model organisms is scarce and, therefore, requires development. We highlight that only 59% of the unigenes were annotated in at least one of the seven databases used, and of the 79 unique DEGs identified, only 27 were annotated against GO. This result shows the knowledge gap on the subject. Furthermore, several genes that were matched against databases were genes with unknown functions, and few studies have been conducted using transcriptomics to evaluate gene dysregulation caused by MeHg. Clearly, the lack of functional annotation of transcriptomes from non-model organisms is limiting the development of the mechanistic understanding of complex traits [67]. However, the proteomes of three turtles (*Chelonia mydas*, *Pelodiscus sinensis*, and *Chrysemys Picta belli*) have already been published, and there are 37 turtle genomes in the process of assembly, 10 of which also have been published, representing important progress for the understanding of these chelonians.

For these reasons, our study represents an important advance of DEGs analysis in the understanding of the toxicity mechanisms by which MeHg affects cellular homeostasis and of the adaptive response of loggerhead sea turtle RBCs. Adaptive responses to MeHg toxicity appear to involve complex polygenic processes. Our findings were consistent with this and reveal that several functionally distinct genes were dysregulated in response to stress produced by MeHg.

In the comparisons made to determine the differential expression of genes between Gc–Gs1, Gs1–Gs5, and Gc–Gs5, the greatest dysregulation occurred in downregulated genes (44 genes) related to the response to cellular stress, signaling, transcription, calcium metabolism, and transport across the membrane, and in upregulated genes (39 genes) involved in the response to stress, lysosomes, mitochondria, regulation of the cell cycle, metabolic processes, and transcription and translation (Table S5).

These results demonstrate that RBCs in Gs1 generally expressed fewer genes, but their response to MeHg was more pronounced (40 dysregulated genes) than RBCs in Gs5 (12 dysregulated genes). The identification of changes in gene transcription can contribute to the identification of molecular events that are in the process of initiation or to the identification of the sequence of molecular events that can lead to cellular dysfunctions, as well as the identification of toxicity markers [29,68]. Studies are needed at lower and higher MeHg concentrations and longer and shorter exposure times.

### 4.1. Differential Expression of Oxidative Stress Indicator Genes

Oxidative stress produced by an increase in reactive oxygen species is one of the earliest responses of RBCs to MeHg toxicity [32]. In this study, when comparing the DEGs between Gc–Gs1, Gs1–Gs5, and Gc–Gs5, the dysregulation of 12 oxidative stress indicator genes was observed: eight upregulated genes (Sgk1, GDP1, HEX_A, ATG5, MKNK1, ZDHHC16, CEP250, and ITGAX) and four downregulated genes (BRD1, DCUN1D2, KPNA6, and SAMD9) (Table 3). These 12 DEGs demonstrate oxidative stress.

The Sgk1 and MkNk1 (Table 3) genes were upregulated. The proteins that these genes encode are linked to various cellular processes, including survival and the response to cellular stress. These proteins carry signals from the cell membrane to the nucleus and are activated in response to environmental, osmotic, or oxidative stress and DNA damage [69]. Everything indicates that these genes in the RBCs are upregulated as a response to stress produced by MeHg to counteract or minimize the toxicity. Overexpression of the Sgk1 gene has been reported to regulate nitric oxide production, protect cells against ROS, and inhibit apoptosis [70]. Although the expression of Sgk1 was not high, its presence denotes the appearance of ROS and, apparently, cellular damage. The significant overexpression of the Atg5 gene supports our hypothesis, as this gene exerts a cytoprotective role in various animal species, making the autophagy process more efficient and reducing oxidative stress during exposure to MeHg [71].

In this study, we found that the expression of Atg5 shows that there is cellular damage in RBCs and its overexpression produces compensation. In mice, the silencing of Atg5 in cells exposed to acute oxidative stress has produced the overexpression of cytokines, which generate inflammation and cellular apoptosis [72]. These findings support the important role this gene plays in RBCs homeostasis. To counteract oxidative stress, the overexpression of other anti-apoptotic genes has been identified, such as Sgk1 and ZDHHC16 (Table 3). The ZDHHC16 gene responds to the stress generated by DNA damage and participates in palmitoylation, a post-translational modification of histone proteins in which the ZDHHC16 enzyme adds a palmitate moiety to specific cysteine residues in RBCs. Palmitoylation affects chromatin remodeling, the structure of DNA, and eventually triggers the activation of regulatory genes that contribute to DNA repair and, therefore, prevent its apoptosis [73,74].

Likewise, we identified the overexpression of the GPD1 gene. The enzyme encoded by this gene reduces dihydroxy-acetone phosphate to glycerol-3-phosphate (G-3-P), reducing in turn one mole of NAD to NADH [75]. Shen et al. [75] described how GDP1 deficiency, under stress conditions, produces the loss of the ability to achieve NADH/NAD balance and, therefore, a constitutively increased level of ROS. However, it is known that GPD1 is involved in a mitochondrial redox shuttle, which serves as a link between the cytosol and the mitochondria, and that a balanced cellular redox state is obtained due to this gene [76]. The overexpression of the GPD1 gene in RBCs is caused by high external osmolarity (MeHg solute), which causes increased synthesis and intracellular accumulation of glycerol [77]. The GPD1 enzyme translocates G-3-P to the inner mitochondrial membrane, which serves as a redox valve to eliminate excess reducing power. In this way, a high NADH/NAD balance remains [76,78]. This response is fundamental for the cell, not only for the control of the redox balance of metabolism, but also, and very importantly, for the preventive management of oxidative stress [75].

Another downregulated gene in the RBCs that is also related to oxidative stress is KPNA6, which is a transport adapter between the nucleus and the cytoplasm and is related to the regulation of cytokine production involved in the inflammatory response to stress [79]. Dysregulation of the KPNA6 gene has been reported to be closely related to nuclear erythroid factor 2 (Nrf2), a skillful regulator of cellular redox homeostasis that regulates the expression of more than 200 genes involved in antioxidant defense [80,81] and, with the Keap1 signaling pathway, promotes the transcription of a large number of genes encoding antioxidant enzymes, detoxification, and xenobiotic transporters as an adaptive response to oxidative stress [79,80].

Nrf2 activation is negatively regulated by Keap1, which exports the Nrf2 transcription factor from the nucleus to the cytoplasm [79,82]. The upregulation of KPNA6 generates the nuclear entry of Keap1 and this neutralizes the signaling of Nrf2 transcription, while the downregulation of the KPNA6 gene decreases the entry of Keap1 to the nucleus and, in this way, Nrf2 triggers adaptive transcription of genes that control oxidative stress [79,83]. We found that the KPNA6 gene is downregulated in loggerhead turtle RBCs and, therefore, Nrf2 activates the expression of the antioxidant enzymes of the cascade of the antioxidant response element (ARE) [83]. This was observed when analyzing the relative expression of the genes (in FPKM). The transcription factor Nrf2 presented a basal expression in all three treatments (FPKM: Gc = 19.7, Gs1 = 18.2, and Gs5 = 17.6). These findings support the idea that KPNA6-mediated nuclear import of Keap1 plays an essential role in modulating the Nrf2/Keap1 pathway and maintaining cellular redox homeostasis [83].

The antioxidant defense systems are essential to neutralize high levels of ROS, which can cause irreversible damage to cells. In this way, cells have antioxidant molecules and detoxifying enzymes that can control free radicals. GSH is the most abundant antioxidant small molecule. Detoxifying enzymes include SOD, GPx, glutathione-S-transferases (GSTs), CAT, glutathione reductase, glutamate-cysteine ligase (GCL), NAD (P) H: quinone oxidoreductase (NQO1), heme oxygenase-1 (HO-1), and other phase II detoxifying enzymes [2,13]. These molecules were not dysregulated, as they did not change in their expression levels. However, the relative expression levels (FPKM) of these genes were much lower than those presented by the HSP70 and ferritin genes (Gc: 10,421 and 5221, Gs1: 10,513 and 5151, and Gs5: 8047 and 5281, respectively).

Heat shock proteins serve many functions as molecular chaperones and folding guide proteins and by preventing protein build-up. During events such as oxidative stress, the expression of heat shock proteins is known to increase considerably [84]. In general, heat shock proteins are activated to stimulate a pro-survival response during oxidative damage [85]. For its part, ferritin acts as a cytoprotective agent that inhibits oxidant-mediated cytolysis in direct relation to its intracellular concentration [86,87]. This idea is crucial to understanding the loggerheads’ response, as we will discuss later.

### 4.2. Lysosomes and Regulation of Autophagy

Low concentrations of MeHg induce autophagy [88], which is mainly due to the association of Atg5 and p53 [89]. In addition to the dysregulation of Atg5 that was already discussed above, in the lysosome and autophagy pathway we found five dysregulated genes: HEX_A, MANB, AP-4, GALNS, and SLC38A9 (Table 3).

We identified the following upregulated genes: HEX_A, which catalyzes the degradation of GM2 gangliosides to GM3 gangliosides in lysosomes [90], molecules which are found in the plasma membrane [91]; MANB, a glycosyl hydrolase that degrades polysaccharides [92]; GALNS, another lysosomal hydrolase that degrades proteins such as glycosaminoglycans, keratan sulfate, and chondroitin-6-sulfate [93]; and AP4B1, which is involved in the generation of vesicles and, in the charge selection, controls the vesicular transport of proteins in different traffic pathways and contributes to the spatial control of autophagy [94]. Apparently, the erythrocyte membrane generates lipids and carbohydrates as a result of ROS-triggered events, such as lipid peroxidation. The dysregulation of the HEX_A and MANB genes could be present in the RBCs, as these two hydrolases execute their task of recycling these molecules through the lysosomal pathway. For their part, the GALNS and AP4B1 genes are downregulated in RBCs and are not part of the response against oxidative stress generated by MeHg.

We found that the SLC38A9 gene, which plays a role as an amino acid sensor upstream of mTORC1 for asparagine, arginine, glutamine, histidine, and lysine, was downregulated [95]. SLC38A9 regulates the activity of mTORC1, an integrator of environmental and hormonal signals, detecting the availability of amino acids, glucose, and cholesterol to initiate growth. Nonetheless, its action mechanism is not clear [95]. Interestingly, SLC38A9 allows the activation of mTORC1 by cholesterol through the recruitment of the NPC1 protein, which is an inhibitor of mTORC1 in cholesterol deficiency [96]. When SLC38A9 fails to activate mTORC1, the anabolic metabolism is suppressed and autophagy is activated [96].

It is clear that the RBCs in the culture medium in which the bioassay was carried out did not have the necessary environmental and hormonal signals for growth. Thus, the activity of the SLC38A9 gene was decreased and its signaling mechanism remained inactive; therefore, RBCs did not reproduce, nor did mTORC1 activation occur [97]. It is important to understand how the SLC38A9 protein identifies and transports amino acids, how it activates or deactivates mTORC1, and how it detects other environmental signals. This topic represents an important area for future research.

### 4.3. Cytoskeletal Stability and Cell Cycle

The cytoskeleton is involved in cell movement and division and is one of the primary targets of MeHg inside the cell. Specifically, it fragments microtubules, disrupting networks that are necessary to perform important biological functions [98]. Vogel et al. [98] assembled in vitro microtubules from the bovine brain and used raw microtubules from the brain of rats. They found similar results in both models: at concentrations of 1 × 10^−5^ M of MeHg, the depolymerization of the microtubules began, reaching the total inhibition of polymerization at a concentration of 3 × 10^−5^ M MeHg. They also observed that 15 MeHg molecules had been attached to the 15 sulfhydryl groups that tubulin (proteins that makeup microtubules) has. This response is what determines the fragmentation of microtubules [98].

The stability of the cytoskeleton of cells is dependent on spectrins and RhoGTpases [99,100]. Spectrins are important proteins of the cytoskeleton because they help to maintain the integrity of the membrane and its morphology, and they participate in the transport of organelles, as well as in the establishment of polarity in RBCs [100]. RhoGTPases are important regulators of the organization of the actin cytoskeleton and their activation is necessary to maintain strong focal and cellular adhesion between cells [99].

We evidenced the upregulation of the spectrin SPTAN1 and the ARHGAP20 RhoGTPase genes. It is highly probable that, as a primary toxicity effect, MeHg initiated an attack on the cytoskeleton of the RBCs. This fact affects important functions, such as the mobilization of secretion and excretion vesicles, displacement of organelles, and intracellular transport of substances, as well as cell division (mitosis and meiosis) [101,102,103]. As an adaptive response, the increased expression of the SPTAN1 and ARHGAP20 genes inhibits microtubule fragmentation and stabilizes the plasma membrane of RBCs [104].

Other genes that regulate the cell cycle were also upregulated in the RBCs. This was the case with CEP250, UHRF2, and CTBP1 (Table 3). The CEP250 gene is related to the positive regulation of the G2/M transition of the mitotic cell cycle and participates in the biogenesis of the centriole and its duplication, the assembly of the spindle-kinetochore, the cell polarity, and the signaling mechanisms of the cell cycle checkpoint [105]. UHRF2, an ubiquitin involved in epigenetics, is closely related to several cell cycle proteins, such as cyclins (A2, B1, D1, and E1), CDK2, and pRb. Furthermore, UHRF2 is involved in the ubiquitination of the cyclins D1 and E1 [106]. UHRF2 is involved in epigenetic regulation by associating with DNMT, G9a, HDAC1, H3K9me2/3, and hemimethylated DNA. The protein encoded by the CTBP1 gene promotes cell cycle progression and has antiapoptotic activity given by the regulation of p53 activity [106,107,108]. Both UHRF2 and CTBP1, as well as Atg5, associate with p53 (tumor suppressor protein), but it is UHRF2 that produces the ubiquitination of p53 in vivo and in vitro [106].

Given that UHRF2-CTBP1 and p53 are all involved in cell cycle regulation, the hypothesis that this is a new signaling pathway in the cell must be studied [109,110]. Possible adaptive reactions to MeHg toxicity in the RBCs can be deducted from the activities of UHRF2 and CTBP1, such as changes in DNA (epigenetic methylation), and from damage to specific proteins of the cell cycle for which the final objective would be apoptosis. However, the upregulation of UHRF2 and CTBP1 involves, on the one hand, protecting the cell from errors in the folding of guide proteins and, on the other, preventing the accumulation of proteins and apoptosis [109,110]. The ubiquitination of proteins for their recycling is an important cellular adaptive response to regain homeostasis during the stress created by ROS produced by MeHg.

Regarding the CEP250 gene, it showed a relatively low upregulation in the RBCs. Probably, this was due to the fact that the RBCs did not have conditions for their reproduction in our bioassay. This adaptive response against MeHg demonstrates cellular damage at this level.

### 4.4. Alteration of Calcium Homeostasis and Mitochondria

One of the most widely documented effects produced by MeHg is related to glutamate-facilitated excitotoxicity, a mechanism that causes excess intracellular Ca^2+^ [111]. The cytoplasmic Ca^2+^ concentration is regulated in RBCs because calcium is a very important element in metabolic and intracellular signaling regulation [111]. In primary cultures of rat astrocytes exposed to concentrations of 1.5 and 10 μM, MeHg produced an increase in the permeability of the mitochondrial membrane, alterations in the glutamine/glutamate cycle, and increased ROS formation [112]. Atchison and Hare [113] reported that MeHg induced the disruption of intracellular Ca^2+^ regulation, blocking of voltage-dependent Ca^2+^ and Na^+^ channels in the plasma membrane, ultrastructural changes, and accumulation of MeHg within mitochondria. At the in vitro level, MeHg inhibits several mitochondrial enzymes and depolarizes the mitochondrial membrane, subsequently reducing ATP production [113].

It has been established that in vitro cell exposure to MeHg generates a Ca^2+^ overload in the cytoplasm, mediated by excitatory amino acid receptors (N-methyl D-aspartate (NMDA) and non-NMDA types) and by Ca^2+^ channels [114,115]. The excess of intracellular Ca^2+^ is distributed between the mitochondria and the smooth endoplasmic reticulum (ER). Mitochondria show low affinity and high capacity to transport Ca^2+^, while the ER has high affinity and low capacity to transport Ca^2+^ [116,117].

The RBCs presented three dysregulated genes: MFF, ATP13A1, and MSTO1. MFF regulates the fission of mitochondria in association with Drp1 [118]. Everything seems to indicate that the intracellular transport of Ca^2+^, when there is an imbalance increasing its intracellular concentration, initially produces pressure for the ER and the mitochondria to absorb part of the Ca^2+^ and avoid apoptosis. However, this triggers the fragmentation of the mitochondria via fission by MFF and the exit of cytochrome C from the mitochondria, which generates greater fission and apoptosis [118]. On the other hand, ATP13A1 is downregulated. Although little is known about its physiological function and properties, it is located in the ER and its function has been linked to calcium homeostasis [119].

The MSTO1 gene is also poorly studied. A mitochondrial location is suggested where mitochondrial morphology (via fusion) and distribution are regulated [120,121]. Fission should cause mitochondrial fusion almost immediately [121]. It is possible to hypothesize that the poor transcription of the MSTO1 gene does not stabilize the mitochondria, so fusion, which would eventually restore mitochondrial form and functions, does not occur. Mitochondrial fission and fusion appear to be regulated by complex molecular processes [120,121].

### 4.5. Regulation of Transcription

MeHg can cause DNA damage by oxidation and by its affinity with macromolecules [122]. Some studies have observed that MeHg produces DNA strand breakage, chromosomal aberrations, micronuclei, and decreased DNA repair [123,124]. However, these consequences are dose-dependent. It is important to note that mitochondrial DNA can also be affected, accentuating the appearance of damage [125].

In an interesting study, Wyatt et al. [125] exposed the *C. elegans* genome to low concentrations of MeHg and HgCl_2_ (1 mM), observing minor DNA damage; suddenly, however, a higher concentration of MeHg (5 mM) decreased the damage compared to controls. This result is in agreement with our findings in this study, where Gs1 expressed 11% fewer genes than Gs5. In the same way, we found evidence of the dysregulation of a good number of genes related to transcription and translation (22 and 4 genes respectively), with the downregulated genes being of greater quantity than the upregulated genes (15 and 11 genes respectively). It is important to note that the highest number of dysregulated genes was present in the Gc–Gs1 comparison (14 genes) and the lowest in the Gc–Gs5 comparison (three genes, less damage at higher concentration). Between Gs1 and Gs5, an intermediate number of genes (11 genes) were dysregulated.

Four dysregulated genes related to the regulation of transcription and gene silencing were detected [126]. Three of them were downregulated: the ZNF280D, PHF20L1, and ZC3H7A genes. ZC3H7A has functions related to post-transcriptional regulation, which could explain the decrease in gene expression [127]. MeHg has been found to induce repression of the chromatin structure in the promoter region of genes by inhibiting its expression [127], so the downregulation of these three genes, which also regulate the expression of other genes, is not strange. ZNF280D, PHF20L1, and ZC3H7A genes are downregulated at low concentrations of MeHg (Gs1) and, as observed at this concentration, there was a significant reduction in gene expression (11% lower than in Gs5). This could explain the decrease in the general expression of genes of Gs1.

In contrast, the PIAS2 gene was upregulated. The enzyme that encodes the PIAS2 gene contains two structural motifs (finger ring and SUMO binding) and one domain (SAP domain) that activate or repress the Elk-1 transcription factor, which depends on the MAPK pathway [128,129,130]. The Elk-1 transcription factor has been shown to bind to the promoters of almost 1000 genes, including IE genes (genes for rapid response to growth factors and other stimuli), genes encoding the basal transcription machinery, spliceosome components, and ribosomal proteins [129]. PIAS2 is a transcriptional co-regulator that activates or represses the transcription of at least 60 proteins. Among the transcription factors that regulate PIAS2 are Jak/STAT and NF-kB [131,132]. The expression of PIAS2, as is clear from this information, is tremendously important in the response to oxidative stress. While in Gs1 the PIAS2 gene was downregulated, and therefore the genes of the Elk-1, Jak/STAT, and NF-kB pathways were not expressed, in Gs5 the PIAS2 gene was upregulated and the pathways were activated. This finding could explain, in part, the reduction in gene expression in Gs1 compared to Gs5 and the difference between Gc and Gs5.

At the same time, the transcription factor gene SOX6 participates in pre-mRNA splicing [132], stimulates cell proliferation, and facilitates the maturation of RBCs [133]. Repression of this transcription factor has been reported to increase globin levels [134,135]. The dysregulation of the SOX6 gene may explain why the RBCs of Gs1, where this gene was downregulated, presented higher levels of alpha hemoglobins, compared to Gc and Gs5 (FPKM: 18.4, 13.6, and 15.1, respectively), as well as of beta hemoglobin (FPKM: 43.4, 14.2, and 29.5, respectively). However, they did not present differential expression or significant differences (Kruskal-Wallis Hb alpha *p* = 0.29 and Hb beta *p* = 0.24).

Ancora et al. [136] showed that Hg binds preferentially to the thiol groups of hemoglobin. Working with dolphins, they exposed blood samples to 0.1 mM MeHg. After a few minutes, 98.1% of the MeHg was in the RBCs, 1.3% in the plasma, and 0.6% in the plasma membrane. It is important to highlight that hemoglobin (Hb) interacts with the carbonic anhydrase-1 protein, the alpha hemoglobin stabilizing protein (AHSP), and Prdx2, with which it forms high-molecular-weight complexes, attenuating the formation of ROS and, in this way, protecting RBCs from oxidative stress [137]. Furthermore, the rate of thiols per hemoglobin tetramer in turtles is as low as in humans (5.6 and 5.8 respectively) compared to crocodiles (16), chondrichthyans (10), birds (9.2), and amphibians (7.2). Therefore, we hypothesize that, as loggerhead turtle hemoglobins are not very reactive, destabilization of their structure by mercuric ions is less possible.

We infer that, in Gs1, MeHg alters the expression of the alpha hemoglobin in the RBCs due to the downregulation of the SOX6 gene, triggering the upregulation of the alpha and beta hemoglobin, resulting in a greater production of ROS and, thus, reducing the overall expression of genes. It is not clear why the SOX6 gene is upregulated in Gs5 and downregulated in Gs1 yet, the only difference between the two groups was the concentration of MeHg.

### 4.6. Analysis of the Relative Expression (FPKM) of the Cysteines and Methionines, Glutathione, Selenocompounds, and Peroxyredoxins Metabolic Pathways

MeHg is an electrophile that regularly attacks nucleophilic groups (thiols and selenoles) [5]. Selenoproteins are the main target for mercury in thioredoxin, peroxiredoxin, glutathione-glutaredoxin, and other selenoprotein (P, K, and T) systems [2]. Mercury binds to selenocysteines in these proteins, inhibiting their function and altering their cellular redox environment, resulting in glutathione excitotoxicity, alteration of calcium homeostasis, damage to mitochondria, lipid peroxidation, deterioration in protein repair, and apoptosis [2,138]. Furthermore, mercury has high affinity for Se, which leads to the depletion of its reserves and, therefore, to the inhibition of selenoprotein synthesis [138].

**Figure 9:**
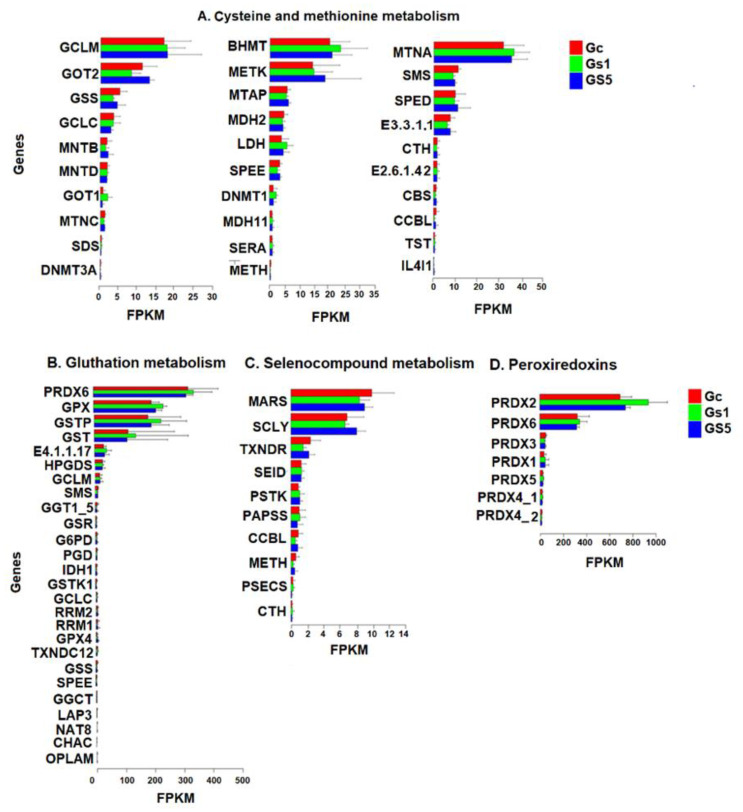
Relative gene expression (FPKM) in loggerhead turtle RBCs in different metabolic pathways. (**A**) cysteine-methionine metabolism, (**B**) metabolism of glutathione, (**C**) metabolism of selenocompounds, and (**D**) metabolism of peroxiredoxins.

The analysis of DEGs carried out in this study found that the enzymes that represent the first line of the antioxidant machinery in response to MeHg were not dysregulated. We did not find dysregulation in genes that encode selenoproteins or thiol-proteins. However, to find out which genes were being expressed, we analyzed metabolic pathways, and we detected 397 metabolic pathways in which all unigenes participate. Among these, we found the metabolisms of cysteines and methionines, glutathione, selenocompounds, and peroxiredoxins. Figure 9 shows the relative expression (FPKM) of the enzymes that are part of the metabolism of cysteines and methionines (Figure 9A). In this pathway, 30 genes were expressed, within which spermine synthase (SMS) was the only gene that presented significant differences (*p* < 0.05) between Gc and Gs1, showing higher relative expression in Gc than in Gs5 and Gs1. SMS is a polyamine with antioxidant and anti-inflammatory properties that have been reported to significantly inhibit the production of nitric oxide (NO), prostaglandins, and cytosines [139], and reduce intracellular MDA levels. This indicates that the RBCs have a constitutive expression of SMS, speD, and E3.3.1.1, all expressed in the pathway of cysteines and methionines, that would play a role in the initial response to oxidative stress generated by MeHg.

In glutathione metabolism (Figure 9B), the relative expression of 26 genes was identified, with high expressions (FPKM = between 100- and 300-fold) of PRDX6, GPX, GSTP, and GST, important enzymes in the response to oxidative stress. However, no significant differences were identified. In all these genes, the expression was always higher in Gs1 (Figure 9 B, green bar). A constitutive expression of these enzymes appears to be present here as well. Reischl [140] found that the glutathione concentration in RBCs of the tortoise *Prynops hilarii* was 1.9 ± 0.2 mM. This result provides grounds to think that the high content of -SH groups must be part of a redox buffer, an output antioxidant to counteract ROS and oxidative stress.

In the metabolism of selenocompounds (Figure 9C), the relative expression of ten genes was identified, without presenting significant differences. The MARS and SCLY genes showed higher relative constitutive expressions (FPKM = between 8 and 10) than the other enzymes of this pathway. Finally, we analyzed the relative expression of peroxiredoxins (Figure 9D), in which only the PRDX2 gene showed significant differences between Gc (FPKM = 694.1) and Gs1 (FPKM = 938.2) (*p* < 0.05). Among the group of peroxiredoxins, PRDX2 is the most efficient enzyme in the elimination of ROS and H_2_O_2_, and the second most abundant protein in the RBCs after hemoglobin [119,120], where it plays a fundamental role in the maintenance of the redox balance and its survival [141,142]. We found that Gs5 produced an intermediate relative expression (FPKM = 741.2). It seems that higher concentrations of MeHg do not encourage the expression of PRDX2 but, on the contrary, slow it down. In general, the genes that code for selenoproteins and that participate in the metabolism of selenocompounds, cysteine, methionine, and glutathione were not affected by in vitro exposure to MeHg. Dysregulation of these genes was not observed, as expected. However, these metabolic pathways show genes with constitutive expressions at high levels, as if turtles are always alert to the appearance of oxidative stress and, in this way, respond adaptively to counteract the harmful effects of toxicity. Krivoruchko and Storey [143], working with adult *Trachemys scripta elegans* turtles and *Chrysemys picta marginata* hatchlings, identified genes that are constitutively upregulated in different organs of these turtles, including: mitochondrial genes encoding electron transport chain proteins, iron storage proteins, serine protease inhibitors, transmembrane solute carriers, receptor proteins of transport and neurotransmission, chaperone proteins, and, most importantly, antioxidant enzymes. These authors proposed “the maintenance of constitutive protection mechanisms”. Turtles maintain a constitutive expression of many proteins that represent the defense against oxidative stress, anoxia, aging, and disease. While expensive in terms of energy consumption, it is very important to provide immediate protection against any metabolic attack.

### 4.7. Correlation between Level of Gene Expression and Enzyme Activity

We found a high statistical correlation between these variables (Figure 8). These analyses have been previously described in other works in which different toxic substances were evaluated in animal species.

In polychaetes (*Perinereis nuntia*), a correlation has been found between the GST gene relative expression levels and GST activity after exposure to a 50 µg L^−1^ dose of copper (Pearson’s correlation, *r* = 0.59–0.85, *p* = 0.001), which was given by the different GST isoforms (GST-omega and GST-sigma) [144,145]. Another study [146] used *Perinereis nuntia* individuals exposed to doses of 50 μg L^−1^ of Cd and found a high correlation between the relative expression levels of the GST-sigma and GST-omega isoforms and the total GST activity (Pearson correlation: *r* = 0.96, *p* < 0.01 and *r* = 0.93, *p* < 0.05, respectively).

Franco et al. [146] studied the effect of oxidative challenges in the relative expression of genes encoding the antioxidant enzymes, Cu/Zn-SOD, Mn-SOD GPx, and CAT, in in vitro muscle cells. They found that the treatment with pro-oxidant paraquat resulted in increases in transcriptional levels of these enzymes and activities. The level of transcription of GPx and CAT increased four- to fivefold and the activities of the enzymes increased two- to threefold. A similar response was presented in the enzymes Cu/Zn-SOD and Mn-SOD. More recently, Zhang et al. [147], working with planarians (*Dugesia japonica*), exposed them to glyphosate and found a correlation between total SOD activity and the relative expression levels of the enzyme Cu/Zn-SOD (Pearson *r* = 0.62).

The results obtained in this correlation show a high relationship between the activity measurements made with classical biochemistry and the relative expression of the genes measured in FPKM. It is important to note that the comparison of more genes could give more robustness to this analysis.

## 5. Conclusions

The toxicology of sea turtles is a relatively new field of research, yet the inclusion of RNAseq, together with bioinformatics and biochemical analyzes, represents a field of study for the present and future of toxicological research for these threatened and endangered animals. The threats posed by contamination by MeHg and other xenobiotics are imminent, and the rigorous study of their action mechanisms is imperative.

We have identified DEGs that affected biological processes in loggerhead turtle RBCs by exposure to MeHg. The downregulated genes were related to cell stress response, signaling, transcription, calcium metabolism, and membrane transport. On the other hand, the upregulated genes were involved in response to stress, lysosomes, mitochondria, regulation of the cell cycle, metabolic processes, transcription, and translation.

At low MeHg concentrations, in Gs1, gene dysregulation was higher compared to Gs5, producing a greater number of down- and upregulated genes. Furthermore, the total number of genes expressed in Gs1 was also lower (−11%) compared to RBCs exposed to a higher concentration of MeHg (Gs5). Everything seems to indicate that low concentrations of MeHg produce greater dysregulation. This hypothesis must be studied in depth to be corroborated.

We found that the RBCs in Gs1 evidenced a greater expression of alpha and beta hemoglobin, which could be related to ROS generation at a higher rate than in Gs5, a fact that could also explain greater dysregulation and lower total gene expression.

According to the analysis of DEGs, a low response of the antioxidant machinery of the early reaction to the toxicity of MeHg can be noted, evidenced by the fact that these genes were not dysregulated. Nonetheless, the expression analysis of the metabolism of cysteines and methionines, glutathione, selenocompounds, and peroxiredoxin enzymes showed a constitutive expression that could be related to “preparation for oxidative stress”, a theory proposed by the biochemist Evaldo Reischl [148], who argued that turtles and other animals have an early response strategy against oxidative stress.

The DEGs identified in this study provide a baseline for further studies on the impacts of MeHg oxidative stress on loggerhead turtle RBCs. We analyzed metabolic pathways using all identified unigenes and detected 397 metabolic pathways in which unigenes participate. This type of analysis has not been widely explored in Hg studies. Transcriptomics and the great advance of bioinformatics support the development of studies in ecotoxicogenomics, providing answers on the molecular mechanisms of toxicity used by MeHg against this sea turtle. There are no previous in vitro or in vivo studies on differential gene expression in RBCs or other tissues in loggerhead turtles. Therefore, it is important to develop additional studies to elucidate the transcriptomic responses of RBCs and other tissues of this turtle when exposed to MeHg.

## Figures and Tables

**Table 1:** Metric and quality of each of the individual samples, the three experimental groups (Gc, Gs1, and Gs5), and the complete transcriptomes of the loggerhead sea turtle, *Caretta caretta*.

Number of Sample	Total Reads	Total Mapped Reads (%)	Total Transcripts	Total Unigens	Mean Length	N50	GC (%)
**T1 (Gc)**	109,774,604	97,540,538 (88.6)	81,393	58,734	994	2628	45.91
**T2 (Gs1)**	110,446,772	100,239,446 (90.8)	61,223	45,142	840	1828	46.86
**T3 (Gs5)**	110,594,626	99,324,656 (89.8)	81,166	58,564	1007	2635	46.87
**T4 (Gc)**	110,454,330	99,989,030 (90.5)	72,525	52,971	1029	2595	47.23
**T5 (Gs1)**	110,371,760	99,933,436 (90.5)	64,043	46,634	893	1996	46.81
**T6 (Gs5)**	110,372,546	99,562,082 (90.2)	77,553	56,342	1045	2676	46.7
**T7 (Gc)**	95,337,750	86,401,432 (90.6)	67,865	50,433	945	2299	46.62
**T8 (Gs1)**	110,614,800	99,565,396 (90)	78,147	75,972	873	2086	46.76
**T9 (Gs5)**	110,518,910	98,688,182 (89.3)	85,833	53,625	878	2194	47
**T10 (Gc)**	110,441,524	96,624,438 (87.4)	108,057	75,428	820	2110	47.03
**T11 (Gs1)**	110,489,326	99,737,464 (90.3)	73,543	58,734	883	2129	47.13
**T12 (Gs5)**	110,350,196	96,400,760 (87.4)	108,082	45,142	800	2035	47.2
**Gc (T1,T4,T7,T10)**	120,987,098	95,049	136,902	95,226	1125	2629	47
**Gs1 (T2,T5,T8,T11)**	115,823,561	71,272	101,265	71,381	1025	2199	47
**Gs5 (T3,T6,T9,T12)**	123,812,263	99,625	148,032	99,809	1110	2616	47
**T. Total (all)**	165,092,512	--------------	192,065	121,933	1444	3520	46.98

**Table 2:** Statistics of the annotation of the composite transcriptome of the loggerhead turtle, *Caretta caretta*, with the number of transcripts that have at least one match with one of the evaluated databases. Some transcripts had multiple annotation results.

Data Base	Number	Percentage
Nr	52,866	43.4
Nt	69,050	56.6
SwissProt	43,994	36.1
KEGG	44,768	36.7
KOG	39,733	32.6
InterPro	38,214	31.3
GO	15,540	12.7
In all databases	11,693	9.6
In five databases	32,467	44.8
General	72,700	59.6
BlastN (*Caretta caretta*)	110,846	90.9
BlastX (Testudines)	97,546	80.5
No annotation information	11,087	9.1
Match with at least one database	110,846	90.9
Total	121,933	100

**Table 3:** Top 25 annotations for the gene ontology of differentially expressed unigenes in loggerhead turtle erythrocytes from the Gc–Gs1, Gs1–Gs5, and Gc–Gs5 comparisons. Upregulated genes (≥1 fold) and downregulated genes (≤−1 fold, *p*-value < 0.01) are shown.

DE	Cellular Functions	Gen ID	Gc–Gs1	Process	Log2FC	*p*-value
**Gc–Gs1**	Stress response, transcription regulator activity, apoptotic process	CL8320.Contig19_All	SGK1	Stress response, regulation of DNA binding transcription, apoptosis inhibitor	1.66	5.53 × 10^−6^
	Stress response, autophagy	CL2170.Contig3_All	ATG5	Nitrosative stress response, negative regulation of ROS, autophagic vesicle formation	7.17	9.83 × 10^−27^
	Stress response	CL5255.Contig3_All	GDP1	Oxidative stress	5.25	2.54 × 10^−12^
	Metabolic process	CL180.Contig4_All	HEX_A	Degradation of GM2 gangliosides	4.92	1.72 × 10^−11^
	Metabolic process	CL192.Contig4_All	MAMB	Beta-mannosidase activity	5.57	1.47 × 10^−14^
	Miscellaneous	Unigene13252_All	AP4B1	Transport of proteins through vesicles to the golgi apparatus and lysosomes	−4.29	2.44 × 10^−8^
	Metabolic process	CL1354.Contig17_All	GALNS	Production of an enzyme called N-acetylgalactosamine 6-sulfatase in lysosomes	−5.35	1.08 × 10^−13^
	Regulation of cell cycle	CL860.Contig14_All	UHRF2	Positive regulation of cell cycle. Proteins marked for destruction	2.04	3.17 × 10^−9^
	Signaling	Unigene60990_All	ATP13A	Cellular calcium homeostasis	−4.78	4.05 × 10^−10^
	Regulation of cell cycle	CL2504.Contig1_All	MSTO1	Regulation of the assembly of mitotic use	1.82	1.75 × 10^−5^
	Transcription regulator activity	CL6965.Contig4_All	ZNF280D	RNA polymerase II cis-regulatory region sequence-specific DNA binding	−4.49	3.61 × 10^−9^
	Transcription regulator activity	CL8179.Contig18_All	PHF20L	Regulator of transcription, gene silencing	−3.37	3.08 × 10^−6^
	Transcription regulator activity	CL439.Contig33_All	ZC3H7A	Posttranscriptional regulation of gene expression, microRNAs and gene silencing	−3.77	0.00651
	Transcription regulator activity	CL7143.Contig1_All	PIAS2	Gene silencing, transcriptional co-regulation in various cell pathways	−5.36	2.14 × 10^−13^
	Transcription regulator activity	CL919.Contig15_All	SOX6	Regulatory transcription, related to neurogenesis and chondrogenesis	−5.19	1.61 × 10^−11^
**Gs1–Gs5**	Stress response	CL2659.Contig5_All	MkNk1	Response to environmental stress and cytokines	4.34	1.29 × 10^−10^
	Stress response	CL1021.Contig3_All	ZDHHC16	Response to stress caused by DNA damage	3.41	7.73 × 10^−6^
	Stress response	CL3160.Contig8_All	KPNA6	Positive regulation of cytokine production involved in inflammatory response	−3.49	6.59 × 10^−6^
	Stress response	CL2820.Contig3_All	CEP250	Transition from G2/M during mitosis	1.12	3.83 × 10^−6^
	Metal ion binding	CL4894.Contig1_All	SLC38A9	Transmembrane amino acid transporter activity	−3.45	1.71 × 10^−7^
	DNA repair	Unigene7038_All	SPATAN1	DNA repair	3.31	7.75 × 10^−6^
	Mitochondria	CL1658.Contig16_All	MFF	Mitochondrial fission	3.71	9.16 × 10^−7^
	Transcription regulator	CL7143.Contig1_All	PIAS2	Gene silencing; transcriptional coregulator in various cellular pathways	3.68	1.59 × 10^−6^
**Gc–Gs5**	Transcription regulator	CL1836.Contig1_All	CTBP1	Regulation of RNA polymerase II, transcription corepressor activity, binding factor	3.9	2.36 × 10^−8^

Log2FC: log2 fold change, positive values represent upregulated genes; negative values represent downregulated genes.

## Supplementary Materials

The following are available online at https://www.mdpi.com/article/10.3390/toxics9040070/s1, Figure S1: title, Table S1. Clean reads quality metrics. Table S2. Functional annotation of genes differentially expressed between Gc and Gs1 groups. Table S3. Functional annotation of genes differentially expressed between Gs1 and Gs5 groups. Table S4. Functional annotation of differentially expressed genes between Gc and Gs5 groups. Table S5. Gene ontology of differentially expressed genes in loggerhead turtle erythrocytes exposed between Gc–Gs1, Gs1–Gs5, Gc–Gs5 for 12 hours. Upregulated genes (≥1 time) and downregulated genes (≤−1 time, *p*-value < 0.01). Figure S1. Electrophoretic profile showing the integrity of the extracted RNA. RIN of the 15 samples evaluated. Figure S2. Length of unigenes. Figure S3. Differentially expressed unigenes. Figure S4A–D. Sperman’s multiple correlation analysis performed between the variables.
